# The sphingosine kinase 2 inhibitor ABC294640 inhibits cervical carcinoma cell growth

**DOI:** 10.18632/oncotarget.23415

**Published:** 2017-12-19

**Authors:** Ling Xu, Longmei Jin, Baohua Yang, Lifeng Wang, Ziyin Xia, Qian Zhang, Jun Xu

**Affiliations:** ^1^ Department of Obstetrics and Gynecology, Minhang Branch, Zhongshan Hospital, Fudan University, Shanghai, China; ^2^ Minhang District Maternal and Child Health Hospital, Shanghai, China; ^3^ Department of Medicinal Chemistry, School of Pharmacy, Fudan University, Shanghai, China

**Keywords:** cervical carcinoma, sphingosine kinase 2, ABC294640, Bcl-2, ceramide

## Abstract

ABC294640 is a specific sphingosine kinase 2 (SphK2) inhibitor. The anti-cervical carcinoma activity by ABC294640 was tested in this study. ABC294640 inhibited *in vitro* growth of the established (C33A and HeLa lines) and primary human cervical carcinoma cells. The SphK2 inhibitor also induced G1-S arrest and apoptosis in cervical carcinoma cells. It was yet non-cytotoxic to SphK2-low human cervical epithelial cells. ABC294640 inhibited SphK activation, causing sphingosine-1-phosphate depletion, signal transducer and activator of transcription 3 in-activation and ceramide production. Bcl-2 is a key resistance factor of ABC294640. Pharmacological Bcl-2 inhibition or Bcl-2 shRNA potentiated ABC294640-induced C33A cell growth inhibition and apoptosis. On the other hand, exogenous over-expression of Bcl-2 attenuated ABC294640's cytotoxicity against C33A cells. *In vivo*, ABC294640 administration inhibited C33A xenograft tumor growth in mice. Co-administration of the Bcl-2 inhibitor GDC-0199 further potentiated ABC294640's anti-tumor activity. Together, we suggest that ABC294640 might have translational value for the treatment of human cervical carcinoma.

## INTRODUCTION

Cervical carcinoma ranks the third most common cancer in young women worldwide. It is an important cause of cancer mortalities [1–3]. The application of HPV-based screening and combination therapy have improved the overall survival of cervical carcinoma [1–3]. Yet, for the patients with advanced, metastatic or recurrent cervical carcinoma, the prognosis is still extremely poor [4]. Groups all over the world are developing novel and molecule-based anti-cervical carcinoma agents [5, 6].

Sphingosine kinase (SphK) is a oncotarget protein for human cervical carcinoma [7] and many other malignancies [8]. SphK is critical for the balance of cellular sphingolipids [9–11]. SphK activation leads to production of sphingosine-1-phosphate (S1P), which is a well-known pro-cancerous lipid molecule. S1P promotes cell survival, cell growth and angiogenesis [12]. Inhibition, depletion or mutation of SphK shall cause production of pro-apoptotic sphingosine and ceramide [13]. Two SphK isoforms, SphK1 and SphK2, have been characterized [14]. The oncogenic function of SphK1 has been well-documented [15]. Literatures have also focused on the potential activity of SphK2 in human malignancies [16–21].

ABC294640 is a novel, specific and potent SphK2 small-molecule inhibitor [16, 17, 22, 23]. Preclinical cancer studies have demonstrated that ABC294640 could possibly inhibit growth of different cancer cells [20, 22, 24]. ABC294640 is a competitive SphK2 inhibitor, which displays chemotherapeutic efficacy in the *in vivo* cancer studies [20, 22, 24]. Currently, ABC294640 is under phase II clinical trials [22]. The potential anti-cancer activity of ABC294640 against human cervical carcinoma cells is tested here.

## RESULTS

### ABC294640 inhibits human cervical carcinoma cell growth

**Figure 1:**
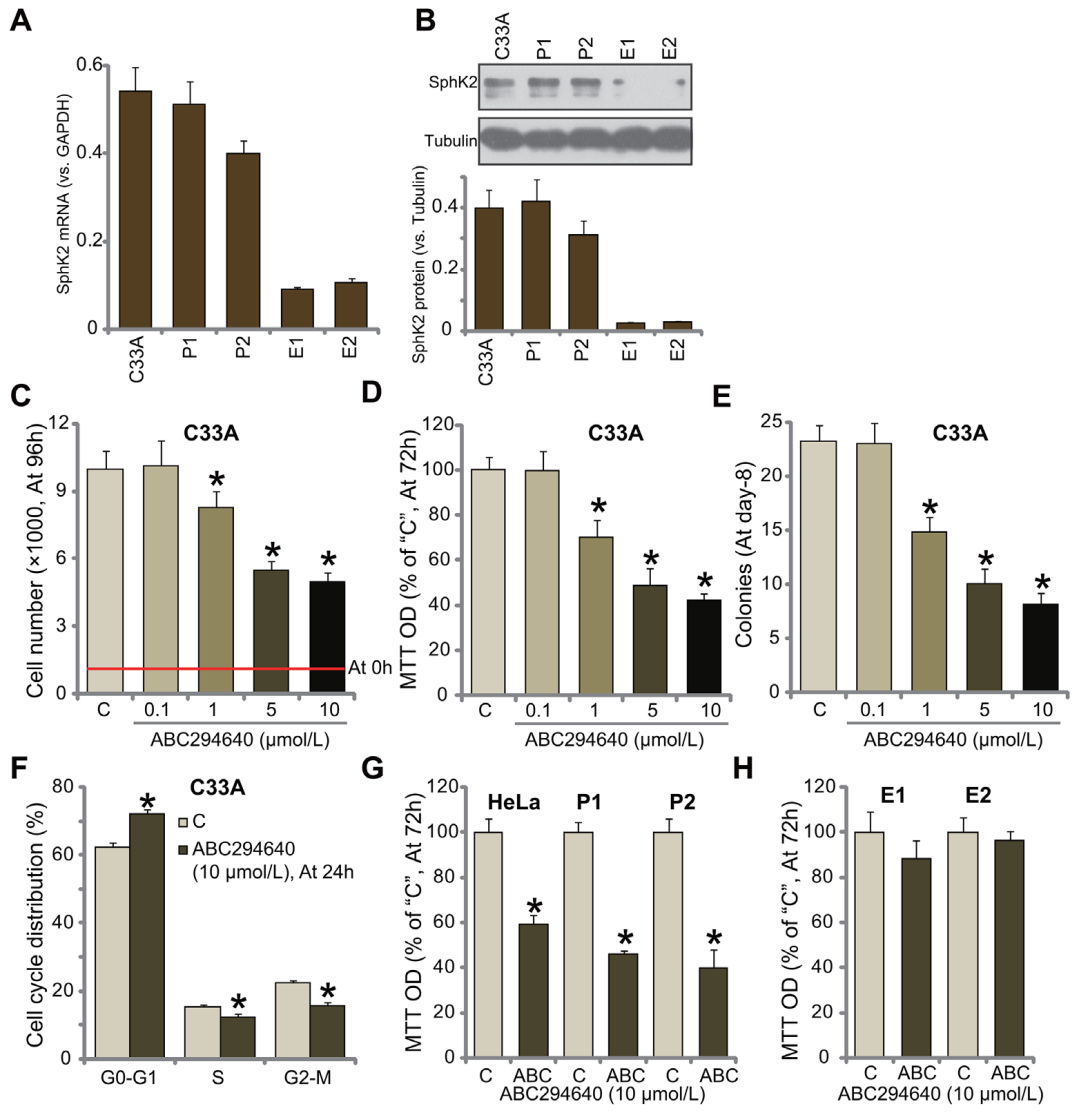
ABC294640 inhibits human cervical carcinoma cell growth mRNA and protein expressions of SphK2 in the established human cervical carcinoma cells (C33A/HeLa), two primary human cervical carcinoma cells (“P1” and “P2”), or primary human cervical epithelial cells (“E1” and “E2”) were shown (**A** and **B**). The above cells were either left untreated (“C”) or treated with ABC294640 at designated concentrations (0.1-10 μmol/L), cells were further cultured for applied time; cell growth was tested by the listed assays (**C-E**, **G** and **H**); cell cycle progression was also tested (**F**, for C33A cells). Data were shown as mean (n=5) ± standard deviation (SD). ^*^p<0.05 vs. “C” cells. Experiments in this figure were repeated three times, and similar results were obtained.

First, we tested the expression of SphK2 in human cervical carcinoma cells. In the current study, two lines of primary human cervical carcinoma cells (“P1” and “P2”) as well as two lines of primary human cervical epithelial cells (“E1” and “E2”) were established. C33A is well-established human cervical carcinoma cell line [25]. The quantitative real-time PCR assay results in Figure 1A confirmed that SphK2 mRNA expression level was high in both primary (“P1” and “P2”) and established (C33A) human cervical carcinoma cells, and its level was relatively low in the primary epithelial cells (Figure 1A). Meanwhile, SphK2 protein was also upregulated in the cancerous cells, as compared to the epithelial cells (Figure 1B).

Cultured C33A cells were treated with ABC294640 at various concentrations (0.1-10 μmol/L). Simple cell counting assay results in Figure 1C demonstrated that ABC294640 inhibited C33A cell growth in a dose-dependent manner. The number of viable C33A cells (at 96-hour) was significantly decreased following ABC294640 (1-10 μmol/L) treatment (Figure 1C). ABC294640, at 1-10 μmol/L, dramatically inhibited MTT optic density (OD) value of C33A cells (Figure 1D). The number of C33A cell colonies was also decreased after ABC294640 (1-10 μmol/L) treatment (Figure 1E). These results suggest that ABC294640 inhibits C33A cell growth.

Results in Figure 1F showed that treatment with ABC294640 (10 μmol/L) in C33A cells also disrupted cell cycle progression, causing G1-S arrest. The potential effect of ABC294640 on other cervical carcinoma cells was also tested. MTT assay results in Figure 1G showed that ABC294640 treatment (10 μmol/L, 72 hours) significantly inhibited growth of primary (two lines, “P1” and “P2”) and the other established HeLa cervical carcinoma cells. On the other hand, the very same ABC294640 treatment failed to inhibit growth (MTT OD) of human cervical epithelial cells (“E1” and “E2”) (Figure 1H). Together, these results suggest that ABC294640 inhibits human cervical carcinoma cell growth *in vitro*.

### ABC294640 induces apoptosis activation in human cervical carcinoma cells

**Figure 2:**
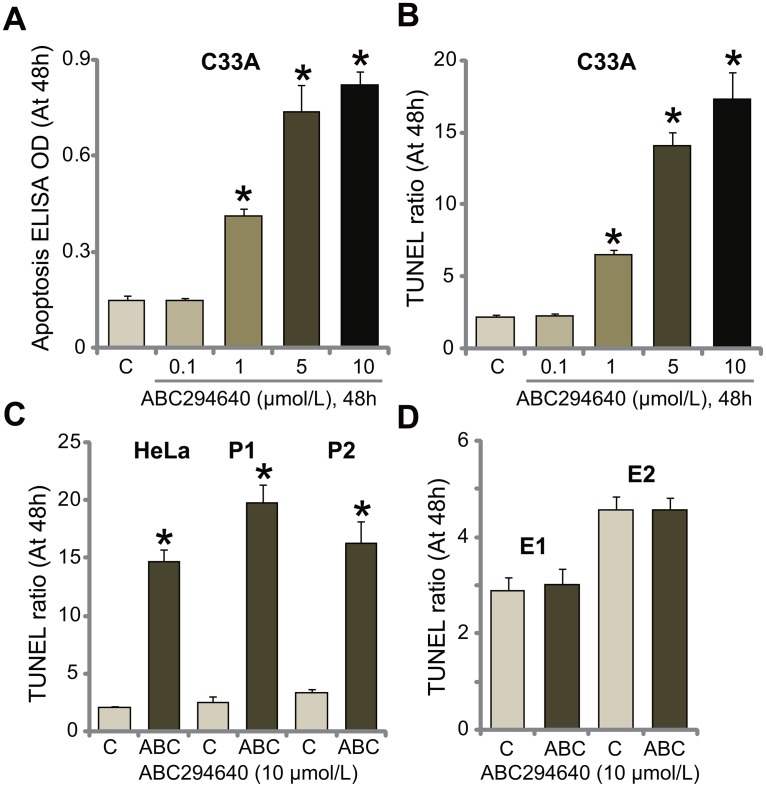
ABC294640 induces apoptosis activation in human cervical carcinoma cells The established human cervical carcinoma cells (C33A and HeLa), the primary human cervical carcinoma cells (“P1” and “P2”), or the primary human cervical epithelial cells (“E1” and “E2”) were either left untreated (“C”) or treated with ABC294640 at designated concentrations (0.1-10 μmol/L), cells were further cultured for applied time; cell apoptosis was tested by the listed assays **(A-D)**. Data were shown as mean (n=5) ± standard deviation (SD). ^*^p<0.05 vs. “C” cells. Experiments in this figure were repeated three times, and similar results were obtained.

SphKs are known anti-apoptosis proteins [14, 26]. Inhibition of SphK could thus induce cell apoptosis [14, 15, 26, 27]. The potential effect of ABC294640 on cervical carcinoma cell apoptosis was tested. As shown in Figure 2A, treatment with ABC294640 in C33A cells dose-dependently increased the Histone DNA apoptosis ELISA OD. Meanwhile, the number of C33A cells with positive TUNEL staining was increased following ABC294640 (1-10 μmol/L) treatment (Figure 2B). These results suggest that ABC294640 provoked apoptosis in C33A cells. In the other established (HeLa) and primary human cervical carcinoma cells (“P1” and “P2”), treatment with ABC294640 (10 μmol/L) similarly induced significant apoptosis activation (TUNEL assay, Figure 2C). Yet, same ABC294640 treatment failed to provoke apoptosis in the primary cervical epithelial cells (“E1” and “E2”) (Figure 2D). These results demonstrate that ABC294640 provokes apoptosis in human cervical carcinoma cells.

### ABC294640 inactivates SphK and STAT3 in C33A cells

**Figure 3:**
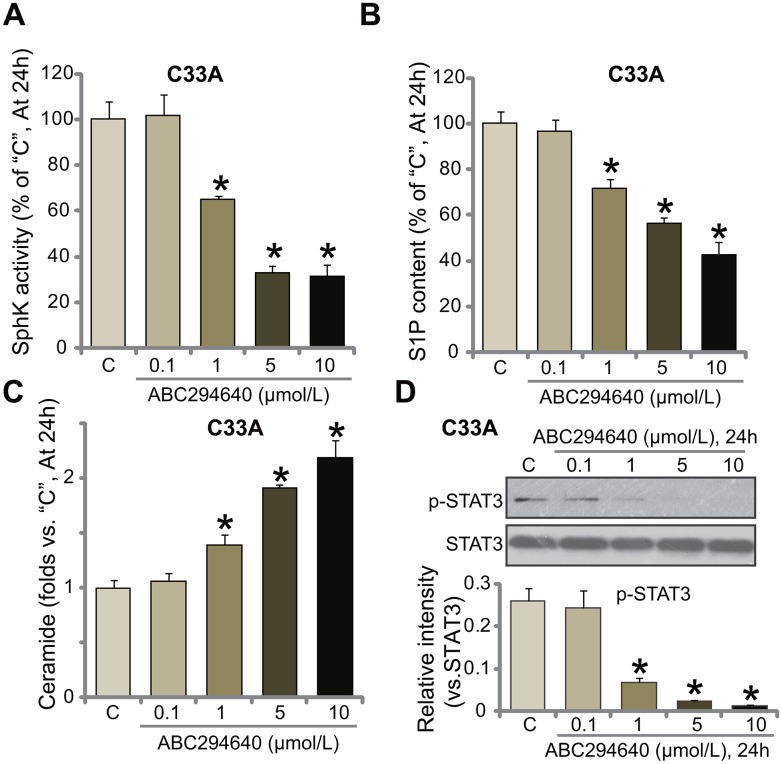
ABC294640 inactivates SphK and STAT3 in C33A cells C33A cells were either left untreated (“C”) or treated with ABC294640 at designated concentrations (0.1-10 μmol/L), cells were further cultured for 24 hours; relative SphK activity **(A)**, S1P content **(B)** and ceramide level **(C)** as well as STAT3 activation **(D)** were examined. p-STAT3 (vs. total STAT3) was quantified (D). Data were shown as mean (n=5) ± standard deviation (SD). ^*^p<0.05 vs. “C” cells. Experiments in this figure were repeated three times, and similar results were obtained.

ABC294640 is the novel and specific SphK2 inhibitor [16, 17, 22, 23], the potential effect of ABC294640 on SphK activity was tested. Using the method described, we showed that treatment with ABC294640 at 1-10 μmol/L significantly inhibited SphK activity in C33A cells (Figure 3A). Consequently, cellular content of sphingosine-1-phosphate (S1P), the pro-cancerous sphingosine [12, 28], was reduced (Figure 3B). On the other hand, the level of pro-apoptotic ceramide was significantly increased (Figure 3C). ABC294640 displayed dose-dependent effect in inhibiting SphK in C33A cells (Figure 3A-3C). At a low concentration (0.1 μmol/L), ABC294640 failed to change SphK activation (Figure 3A) nor S1P/ceramide production (Figure 3B and 3C).

S1P could serve as a key upstream signal of STAT3 (signal transducer and activator of transcription 3) [29, 30], the latter is a key participant in cervical carcinoma progression [31, 32]. In the current study, we showed that STAT3 activation, tested by p-STAT3 at Tyr705, was largely inhibited in ABC294640-treated C33A cells (Figure 3D). Collectively, these results show that ABC294640 inactivates SphK and STAT3 in C33A cells.

### Inhibition or silence of Bcl-2 sensitizes ABC294640 in C33A cells

**Figure 4:**
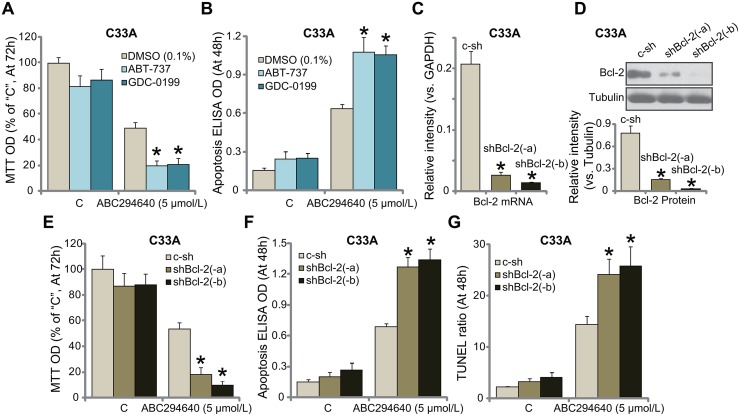
Inhibition or silence of Bcl-2 sensitizes ABC294640 in C33A cells C33A cells were treated with ABC294640 (5 μmol/L), together with/out ABT-737 (200 nM) or GDC-0199 (200 nM), cells were further cultured for indicated time, cell growth (MTT assay, **(A)** and apoptosis (Histone DNA ELISA assay, **(B)** were tested. mRNA **(C)** and protein **(D)** expression of Bcl-2 in stable C33A cells, expressing Bcl-2 shRNA (“shBcl-2-a/-b”, with non-overlapping sequence) or scramble non-sense control shRNA (“c-sh”), were shown; cells were also treated with ABC294640 (5 μmol/L) for indicate time; cell growth (MTT assay, **(E)** and apoptosis (**F** and **G**) were tested. Data were shown as mean (n=5) ± standard deviation (SD). ^*^p<0.05 vs. DMSO (0.1%) cells (A and B). ^*^p<0.05 vs. “c-sh” cells **(C-G)**. Experiments in this figure were repeated three times, and similar results were obtained.

One important objective of this study is to identify possible ABC294640's resistance factors. We focused on Bcl-2. Bcl-2 is well-established anti-apoptosis protein [33–35. Bcl-2 has been recognized as a key chemo-resistance factor [33–35]. Third, Bcl-2 inhibition, mutation or depletion could significantly sensitize the anti-cancer activity by a number of molecule-targeted agents [36–40]. Bcl-2 pharmacological inhibitors were then applied, including the specific Bcl-2 inhibitor GDC-0199 (also known as ABT-199) [41, 42] and the pan Bcl-2 inhibitor ABT-737 [37, 40, 43]. As shown in Figure 4A, ABC294640 (5 μmol/L)-induced C33A cell growth inhibition (MTT OD reduction) was largely potentiated with co-treatment of the Bcl-2 inhibitors, which also dramatically facilitated ABC294640-induced cell apoptosis (TUNEL assay, Figure 4B). These results imply that Bcl-2 inhibition could possibly sensitize ABC294640 in C33A cells.

To exclude the off-target effect of the above inhibitors, *i.e.* ABT-737 [37, 40, 43], we utilized shRNA method to selectively knockdown Bcl-2 in C33A cells. Two distinct Bcl-2 shRNAs (“-a/-b”), with non-overlapping sequences, were applied. Quantitative real-time PCR (“qRT-PCR”) assay results in Figure 4C demonstrated that the applied Bcl-2 shRNA dramatically silenced *Bcl-2 mRNA* in C33A cells. Consequently, Bcl-2 protein expression was also silenced in stable cells with Bcl-2 shRNA (Figure 4D). Remarkably, knockdown of Bcl-2 significantly sensitized ABC294640 (5 μmol/L)-induced cytotoxicity against C33A cells, causing substantial growth inhibition (MTT OD reduction, Figure 4E) and apoptosis (Apoptosis ELISA OD/TUNEL ratio increase, Figure 4F and 4G). Collectively, these results confirm that inhibition or silence of Bcl-2 sensitizes ABC294640 in C33A cells.

### Exogenous over-expression of Bcl-2 de-sensitizes ABC294640 in C33A cells

**Figure 5:**
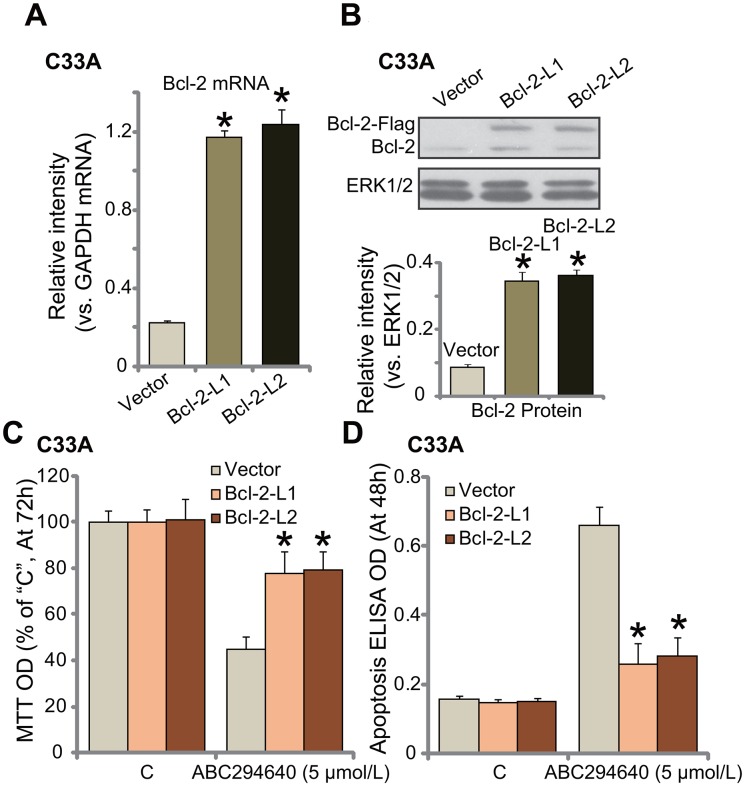
Exogenous over-expression of Bcl-2 de-sensitizes ABC294640 in C33A cells mRNA **(A)** and protein **(B)** expression of Bcl-2 (endogenous and exogenous) in stable C33A cells, expressing the Bcl-2 construct (two lines, “Bcl-2-L1/L2”) or empty vector (Ad-GFP, “Vector”) were shown; cells were also treated with ABC294640 (5 μmol/L) for indicate time; cell growth (MTT assay, **(C)** and apoptosis (Histone DNA ELISA assay, **(D)** were tested. Data were shown as mean (n=5) ± standard deviation (SD). ^*^p<0.05 vs. “Vector” cells. Experiments in this figure were repeated three times, and similar results were obtained.

Next, the Bcl-2 expression vector (a gift from Dr. Sun [38]) was introduced to C33A cells, and two stable cell lines (“L1” and “L2”) expressing the construct were established. As compared to vector control cells, *Bcl-2 mRNA* level was significantly increased in the two stable lines (Figure 5A). Bcl-2 protein was also over-expressed (Figure 5B). Notably, the exogenous Bcl-2 was tagged with Flag (Figure 5B). Remarkably, ABC294640-induced growth inhibition (MTT OD reduction, Figure 5C) and apoptosis (Histone DNA ELISA assay, Figure 5D) were largely attenuated in Bcl-2-over-expressed C33A cells. Thus, exogenous over-expression of Bcl-2 could significantly attenuate ABC294640's cytotoxicity against C33A cells, further confirming the chemo-resistance function of Bcl-2 against ABC294640.

### ABC294640 inhibits C33A tumor growth in nude mice, sensitized with co-administration of GDC-0199

**Figure 6:**
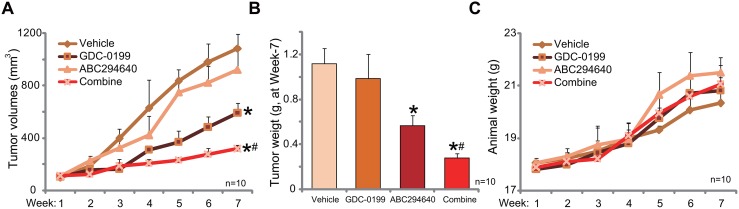
ABC294640 inhibits C33A tumor growth in nude mice, sensitized by co-administration of GDC-0199 C33A tumor-bearing mice were administrated withABC294640 (20 mg/kg, daily, *p.o.,* for 21 days) and/or GDC-0199 (25 mg/kg, daily, *i.p.,* for 21 days); estimated tumor volume **(A)** and mice body weight (deducting tumor weight, **(C)** were recorded weekly for total six weeks. At the end of experiment (week-7), tumors of the each group were isolated and weighted **(B)**. Data were shown as mean (n=10) ± standard deviation (SD). ^*^p<0.05 vs. “Vehicle” (0.375% Polysorbate-80) treatment. ^#^*p*<0.05 vs. ABC294640 only treatment.

At last, the anti-cervical carcinoma activity of ABC294640 *in vivo* was tested. As described, C33A cells were *s.c.* injected to the nude mice. Within 2-3 weeks, when the tumors were established (with 100 mm^3^ in volume), mice were randomly assigned into four groups, with 10 mice per group. Tumor growth curve results in Figure 6A demonstrated that oral administration of ABC294640 (20 mg/kg, daily, *p.o.,* for 21 days) [17] inhibited C33A tumor growth in mice. The tumor volumes in ABC294640-treated mice were significantly lower than those of vehicle control mice (Figure 6A). Remarkably, co-administration of the Bcl-2 inhibitor GDC-0199 (25 mg/kg, daily, *i.p.,* for 21 days) [41, 42] strikingly potentiated the anti-cancer activity by ABC294640, leading to profound inhibition of C33A tumors (Figure 6A). The combined activity was dramatically more potent than ABC294640 single treatment in inhibiting C33A tumors (Figure 6A). The weight of tumors was also lowest in the combination treatment group (Figure 6B). Although, ABC294640 single treatment also decreased the weight of C33A tumors (At week-7, Figure 6B). Notably, treatment with GDC-0199 alone failed to induce significant inhibition on C33A tumor growth (Figure 6A and 6B). Mice body weight, the major indicator of animal health, was not significantly changed by the single or combined treatment (Figure 6C). We also failed to notice any signs of apparent toxicities during the experimental periods. Thus, these nude mice were obviously well-tolerated to the treatment regimens here.

## DISCUSSIONS

The results of this current study proposed that SphK2 could be a novel oncotarget protein and rational therapeutic target for human cervical carcinoma. We demonstrated that SphK2 is over-expressed in primary and established human cervical carcinoma cells. ABC294640, the novel, specific and competitive SphK2 inhibitor, in-activated SphK in cervical carcinoma cells, leading to S1P depletion, ceramide production and STAT3 inhibition. Treatment with ABC294640 induced growth inhibition, G1-S arrest and apoptosis in a panel of established human cervical carcinoma cells. *In vivo*, ABC294640 oral administration at a well-tolerated dose potently inhibited C33A xenograft in nude mice. These results suggest that targeting of SphK2 by ABC294640 could inhibit human cervical carcinoma cells *in vitro* and *in vivo*.

Signal transducers and activators of transcription (STAT) transcription factor family proteins are vital in the regulation a number of key cancerous behaviors, including cell survival, growth, apoptosis-resistance, migration, and angiogenesis [44, 45]. Constitutively-activated STAT3 is often detected in cervical carcinoma, which is associated with tumorigenesis, cancer progression and poor prognosis [44, 45]. It has been previously shown that S1P is essential for production of NF-κB-regulated cytokine IL-6, and subsequent activation of STAT3 [29]. On the other hand, S1P depletion could significantly inhibit STAT3 activation. In the current study, we showed that ABC294640 inhibited SphK activity, causing S1P reduction and downstream STAT3 inactivation. This could be an important reason of the superior anti-cervical carcinoma activity by ABC294640. The underlying mechanisms of STAT3 inhibition by ABC294640 may warrant further investigations.

Another novel finding of this study is that Bcl-2 could be a major resistance factor of ABC294640 in cervical carcinoma cells. Pharmacological inhibition (by adding ABT-737 or GDC-0199) or silence of Bcl-2 sensitized ABC294640-induced anti-C33A cell activity. On the other hand, exogenous over-expression of Bcl-2 de-sensitized ABC294640 in C33A cells. *In vivo*, co-administration of the Bcl-2 inhibitor GDC-0199 significantly potentiated ABC294640-induced anti-C33A tumor activity in nude mice. Therefore, targeting Bcl-2 should sensitize ABC294640's anti-cancer efficiency. Further studies will be needed to explore the mechanism of ABC294640's resistance by Bcl-2. It will be interesting to test this theory in other cancer cells.

Together, we suggest that targeting SphK2 by ABC294640 inhibits human cervical carcinoma cell growth *in vitro* and *in vivo*. Bcl-2 inhibition could further sensitize ABC294640's activity against cervical carcinoma cells. ABC294640 might have translational value for the treatment of cervical carcinoma.

## MATERIALS AND METHODS

### Reagents

ABC294640 was provided by DC Chemicals (Shanghai, China). ABT-737 and GDC-0199 were obtained from Selleck (Shanghai, China). Puromycin was purchased from Sigma Aldrich (St. Louis, MO). The antibodies utilized in this study were from Cell Signaling Tech (Beverly, MA).

### Cell culture

The established human cervical carcinoma cell lines, C33A and HeLa were provided by the Cell Bank of Shanghai Institute of Biological Science (Shanghai, China). Cells were maintained in the DMEM/F12 medium with 10% FBS. Two cervical carcinoma patients administrated at the Minhang Branch, Zhongshan Hospital were consent to provide the tissues. The cervical carcinoma and para-carcinoma epithelial tissues were obtained at the time of surgery. The tissues were separated carefully and were digested for 10 hours at 4°C in dispase. Tissues were cut into 1 mm^2^ pieces and digested in 0.25% trypsin for 15 min at 37°C. Trypsin was then neutralized by FBS (10%). The cells were collected by low speed centrifugation. Primary cancer cells were cultured in the DMEM plus 10% FBS medium. Cultures with 95% epithelial cells were maintained in keratinocyte serum-free medium (KSFM, Invitrogen, Carlsbad, CA). A total of two lines of primary human cervical carcinoma cells, namely “P1” and “P2”, as well as two lines of primary human cervical epithelial cells (“E1” and “E2”), were established. The protocols were approved by the Institutional Review Board and Ethics Committee of Fudan University, and experiments were conducted according to the principles of Declaration of Helsinki.

### MTT assay of cell growth

Cell growth was examined by routine 3-[4, 5-dimethylthiazol-2-yl]-2, 5 diphenyltetrazolium bromide (MTT) assay. Briefly, 5,000 cells per well were plated onto the 96-well plates. After the applied treatment, MTT (5 mg/mL, 20 μL per well, Sigma) was added to the medium for another 2-3 hours. Absorbance at 490 nm of MTT was measured by a microplate reader (Bio-Rad, Basel, Switzerland).

### Clonogenicity assay

C33A cells were initially plated at 10,000 cells/well onto the six-well plate, and were treated with designated concentration of ABC294640. Cells were further incubated for another eight days. The number of colonies was then manually counted.

### Cell cycle distribution analysis

C33Acells with applied ABC294640 treatment were fixed in ice-cold ethanol, which were then washed and stained with 10 μg/mL propidium iodide (PI, Invitrogen) and100 μg/mL RNase (Invitrogen). DNA content was analyzed with a flow cytometer (BD Biosciences, Franklin Lakes, NJ). Cell cycle distribution (G0-G1, S and G2-M) percentage was recorded.

### Fragmented DNA detection by ELISA

Histone-bound DNA was tested via a specific two-site ELISA kit (Roche, Shanghai, China). Histone DNA ELISA OD was recorded at 450 nm was tested.

### TUNEL assay of apoptosis

Following the ABC294640 treatment, cells were subjected to the TUNEL dye assay (Invitrogen). For each condition, at least 200 cells in five random views were counted. TUNEL percentage (vs. total cell number) was recorded.

### Western blotting assay

Lysate proteins were separated by 8-12% of SDS-PAGE gels, which were then transferred to PVDF blots (Millipore, Wuxi, China). After blocked in 10% milk, the blots were incubated with the designated primary and corresponding secondary antibodies. The protein signals were visualized via the ECL detection kit. Quantification of the signal was performed via the Image J software.

### Bcl-2 shRNA

Six distinct lentiviral shRNAs against non-overlapping sequence of human Bcl-2 were designed and synthesized by Genepharm (Nanjing, China). The lentiviral shRNA (10 μL/mL) was added to cultured C33A cells for 24 hours. Afterwards, puromycin (2.5 μg/mL, Sigma) was utilized to select stable cells, which could last for another 8-12 days. Bcl-2 knockdown in stable cells was verified by qRT-PCR assay and Western blotting assay. Of the five tested Bcl-2 shRNAs, two of them efficiently downregulated Bcl-2. Control cells were infected with lentiviral scramble control shRNA (Santa Cruz Biotech).

### Exogenous Bcl-2 over-expression

The Bcl-2 expression vector was a gift from Dr. Sun [38]. The construct was transfected to C33A cells via the routine Lipofectamine 2000 protocol [46]. Puromycin (2.5 μg/mL, Sigma) was added again to select stable cells for another 8-12 days. Two stable C33A cell lines with the construct were established. Bcl-2 expression (endogenous and exogenous) in the stable cells was verified by qRT-PCR assay and Western blotting assay.

### Quantitative RT-PCR

Trizol reagents (Sigma) were applied to extract total cellular RNA, and High Capacity cDNA Reverse Transcription Kit was utilized to synthesis cDNA from 0.5 μg mRNA (per treatment). Power SYBR Green RT-PCR Reagents Kit was used to perform the quantitative real-time PCR (“qRT-PCR”) via the ABI-7500 system (Applied Biosystems, Foster, CA). The primers for *Bcl-2 mRNA* were described previously [47, 48]. The primers GAPDH *mRNA* were also described early [49]. *SphK2 mRNA* primers were described previously [50]. Relative mRNA expression was carried out using 2^−ΔΔCt^ method after normalization to GAPDH.

### Assay of SphK activity and S1P content

Following the indicated ABC294640 treatment, 20 μg protein lysates (per treatment) were incubated with D-erythrosphingosine (5 μmol/L, dissolved in 0.1% Triton X-100, 2 mmol/L ATP, and [γ-32P] ATP) [17]. HCl (1 N) was added to stop the reaction, followed by adding 800 μL of chloroform/methanol/HCl (100:200:1, v/v). After vortex, phases were separated by centrifugation. Radio-labeled S1P was separated by 60 thin-layer chromatography (TLC) on silica gel G60-plates with the described solvent, and phosphate incorporation was visualized and quantified via the scintillation counter (LS-6500, Beckman, Shanghai, China) [51]. The SphK activity was valued as pmol/hour/g protein, which was always normalized to that of control group.

### Ceramide detection

Autoradiography detection of cellular ceramide content was described in detail in previous studies [52, 53]. Ceramide content in the treatment group was always normalized to the control level.

### C33A xenograft assay

All animal protocols in the study were approved by Fudan University's Ethics Board. Animals were provided by the Animal Center of Fudan University (Shanghai, China) and were maintained in routine conditions. The female nude mice (6-8 weeks age, 17.5-18.8 g weight) were subcutaneously (*s.c.*) inoculated with 5 × 10^6^ C33A cells (per mouse, in 0.2 mL DMEM/10 % FBS) into the right flanks. When C33A xenograft volumes reached around 100 mm^3^, mice were randomized into four groups, which were treated as describe in the text. The xenografted tumor volumes along with mice body weights were recorded every week for a total of six weeks. At the end of experiment (week-7), tumors of each group were isolated and weighted.

### Statistics

Data were expressed as the mean ± SD (standard deviation). Comparisons between groups were performed via one-way ANOVA (SPSS 18.0). p values < 0.05 were considered statistically significant.
